# The Rarity of Maxillary Osteomyelitis: Insights From a Unique Case Report

**DOI:** 10.7759/cureus.61202

**Published:** 2024-05-27

**Authors:** Tanishq Kumar, Rajoshee R Dutta, Arihant Singh, Ashok M Mehendale, Kaushal Charan Pahari

**Affiliations:** 1 Medicine, Jawaharlal Nehru Medical College, Datta Meghe Institute of Higher Education & Research, Wardha, IND; 2 Preventive Medicine, Jawaharlal Nehru Medical College, Datta Meghe Institute of Higher Education & Research, Wardha, IND; 3 Maxillofacial Surgery, SB Aesthetics, Gurugram, IND

**Keywords:** chronic suppurative osteomyelitis, infraorbital abscess, sequestrectomy, diabetes mellitus, maxillary osteomyelitis

## Abstract

Maxillary osteomyelitis is a rare bone infection and is rarer to come across with the advent of advanced antibiotic therapies. It is often linked to immunocompromised conditions, namely diabetes mellitus, cancer, and chronic alcoholism, as they increase the chances of developing osteomyelitis. We present a rare case of maxillary osteomyelitis along with an infraorbital abscess in a 32-year-old male patient with uncontrolled diabetes. The patient complained of dental pain, facial swelling, and visual disturbances. The patient was managed with sequestrectomy along with curettage, incision, and drainage of orbital abscess. The patient responded well to surgery and had no complications post-surgery. As radiographic signs may present late, the authors aim to highlight the significance of thorough clinical examination and good patient history. Prompt radical treatment is necessary to avoid any severe consequences.

## Introduction

Osteomyelitis is characterized by the inflammation of the bone and bone marrow manifesting majorly due to infection within the medullary cavity. It swiftly engulfs the haversian system and spreads to the periosteum. Before the widespread use of antibiotics, osteomyelitis of the jaws was indeed a common finding as compared to today's time and era. Various organisms that cause osteomyelitis of jaws, such as Staphylococcus aureus, Staphylococcus epidermidis, Peptostreptococcus, Fusobacterium, and Prevotella have been identified that are capable of causing odontogenic infections. It proved to be fatal with poor patient outcomes. The incidence and prognosis have significantly improved with the advent of antibiotics, advanced oral hygiene, and modern treatment modalities [1].

Nonetheless, in recent years, there have been several reported cases that show an association between osteomyelitis of the jaws and patients with a compromised immune system, namely due to untreated diabetes mellitus, ongoing cancer treatment and poor nutrition [2]. Osteomyelitis affecting the maxillofacial is rare; the mandible is more commonly affected than the maxilla. The possible reason behind this contrast could be due to the cancellous bone along with rich blood supply to the maxilla via collaterals, thus making it less prone to infection [3,4]. Treatment of osteomyelitis requires a multifaceted approach involving the surgical removal of dead bone tissue and the complete eradication of any prevalent pathogenic organisms through different treatment modalities of surgery, antibiotics, and palliative care. Dangerous sequelae of maxillary osteomyelitis can lead to infections in the brain and cranial bones. For optimum patient outcomes, prompt diagnosis and aggressive management involving a multidisciplinary approach are needed to save the patient from dire complications [2]. We hereby report a case of chronic suppurative osteomyelitis of the maxilla and right infraorbital abscess in a 32-year-old patient with an immunocompromised state of uncontrolled diabetes mellitus.

## Case presentation

A 32-year-old male patient, a known case of diabetes mellitus with poor glycemic control along with tobacco and alcohol addiction complained of pain in the right upper molars and premolars, swelling of the right side of the face below the right eye, and retroorbital pain along with difficulty in vision of the right eye for past one month. The patient had consulted a dentist for these complaints, following which, debridement, curettage, and dental extraction were done. However, the patient's initial complaints persisted even after dental intervention. Further complications led to double vision, prompting referral to the regional hospital.

On general examination, submandibular lymphadenopathy was the only positive finding, with a palpable, tender, firm lymph node measuring 1.2 x 0.5 cm. On extraoral examination, facial asymmetry was noted particularly with swelling below the right eye. The swelling was pale red in colour, spherical in shape, 1.5cm X 0.7 cm in size (approximately), smooth in surface, sessile, one in number, non-pulsatile and was associated with partial closure of right eye. The swelling was accompanied by restricted opening of the right eyelid with infraorbital oedema. Restricted gaze and diplopia in the right eye along with normal pupillary reflexes were also observed. Extraoral examination's palpatory findings suggested marked temperature rise and tenderness in the extraoral swelling, along with diffuse swelling. The swelling extended from the infraorbital margin to the zygomatic arch superior-inferiorly and mediolateral from the inner canthus to the outer canthus of the eye.

**Figure 1:**
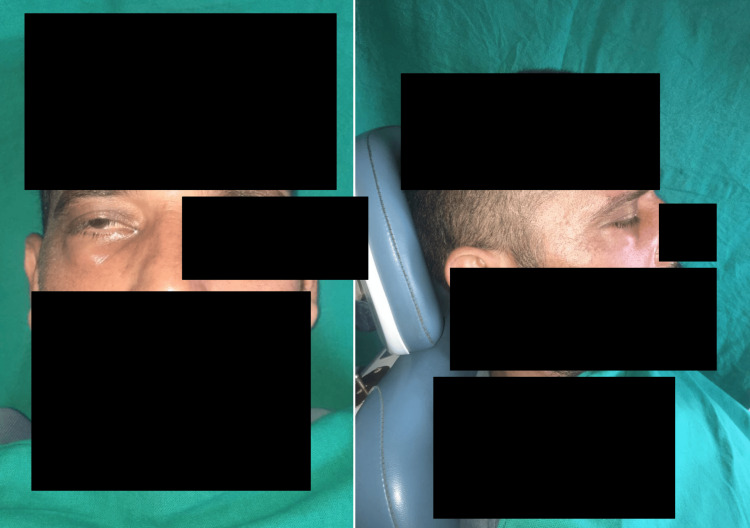
Pre-operative images of the patient

In intraoral examination, soft tissue examination revealed erythematous mucosal lining covering the hard palate on the right side, inflamed mucosal lining in the right vestibule, and unhealed extraction sockets in the maxillary region. Hard tissue examination exposed palatal bone as first premolar to second molar (tooth 14,15,16,17) were extracted with unhealed extraction sockets, pale yellow to light brown exposed bone, and indurated, tender soft tissue with a wooden character. Figure 1 shows pre-operative images of the patient.

**Figure 2:**
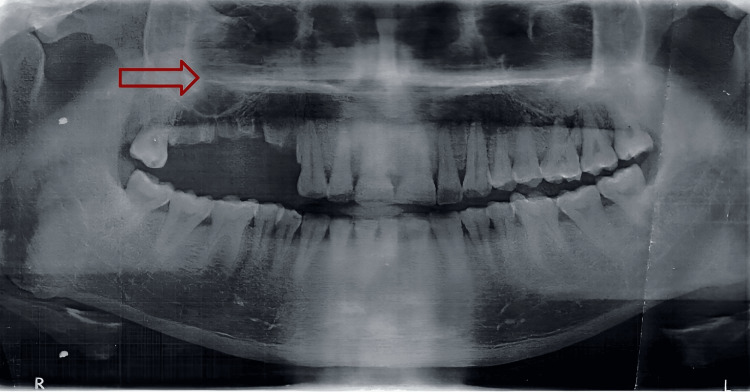
Panoramic radiograph showing a moth-eaten appearance, radio-opaque islands suggesting sequestra, granular densification, and scattered areas of bone densification.

Panoramic radiograph revealed changes in bone density of the affected part. Multiple radio-opaque foci suggesting sequestra are also seen. Irregular and ill-defined changes give the classic moth-eaten appearance. Granular densification and scattered areas of bone densification in the right maxillary region were observed. Marked acute inflammation due to the presence of necrotic bone tissue supported the previously diagnosed osteomyelitis, confirming the diagnosis. Based on the above clinical findings, examinations, and investigations, the diagnosis of chronic suppurative osteomyelitis of the right maxilla and right infraorbital abscess was made. Figure 2 is a panoramic radiograph showing a moth-eaten appearance, radio-opaque islands suggesting sequestra, granular densification, and scattered areas of bone densification.

**Figure 3:**
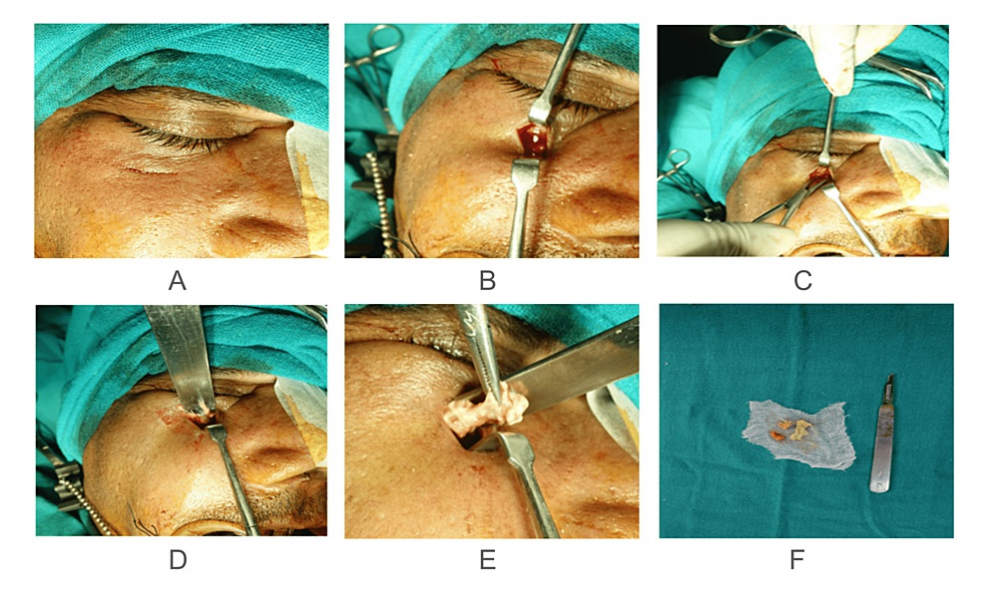
Intra-operative images of the patient A: Painting and draping was done under aseptic conditions; B: Local infiltration was given followed by incision and reflection; C: Curved artery was introduced to gain the access; D: After gaining access the space was reflected; E: Necrosed tissue was removed; F: Image of removed necrosed tissue.

**Figure 4:**
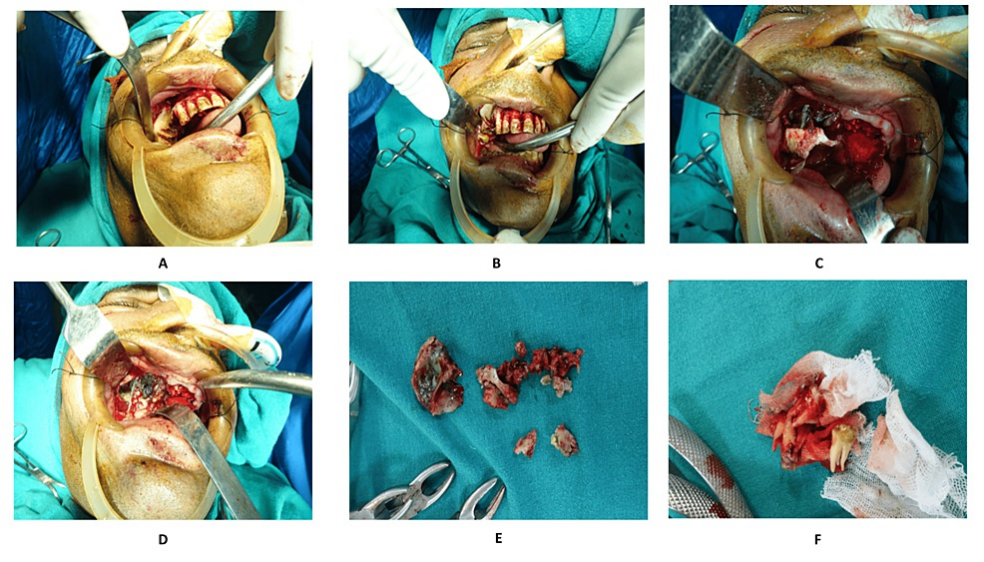
Intra-operative images of the patient along with removed necrotic tissue A: Intraoral view of the site of exposure; B: Soft tissue excision and exposure done; C: Dead and necrotic bone exposed; D: Excision of bone intraoral; E: Excised dead bone with extracted tooth of the affected side; F: Extracted tooth of contralateral side

The treatment plan was an urgent, aggressive surgical intervention comprising of sequestrectomy along with curettage, incision, and drainage of orbital abscess. The treatment was done after ensuring that the patient had adequate glycemic control. The overview of the surgical intervention included painting and draping under aseptic conditions, local infiltration followed by incision and reflection, and a curved artery was introduced to gain access. After gaining access, the space was reflected, and the necrotic tissue was removed. Soft tissue was exposed and excision of dead bone along with extracted tooth of the affected side was done. The patient underwent an uneventful recovery without any complications post-surgery. The patient regularly followed up for six months without any relapse of symptoms. Figure 3 and Figure 4 summarize surgical steps.

## Discussion

The prevalence of conditions like osteomyelitis in today's world of medical advancement is rare, and affecting the maxillofacial part of the bone structure is rarest of rare. In this spectrum of exceptions, osteomyelitis of the maxilla is one of the rare cases that might present in one's outpatient department. It is difficult to diagnose such disease as it manifests under the garb of conditions like infection through the hematogenous route, trauma, and immunocompromised conditions like diabetes mellitus, cancer, and malnutrition state [5,6]. Generally, there are three causes of osteomyelitis of the jaw: traumatic, usually after a road traffic accident or hospital-acquired, rhinogenic origin and odontogenic infections [7].

After pathogen infiltration, there are changes in the blood pH along with changes in capillary permeability. This results in localized edema, production of cytokines, tissue degradation, and poor tissue perfusion, leading to low levels of oxygen at tissue level. Localized increase in pressure, thrombosis in small vessels, and bone degradation. When the medullary cavity gets infected, pressure spike leads to cavity expansion up to the cortex through the Haversian and Volkmann canals. Further, the infection spreads to the subperiosteal space, resulting in periosteal stripping. Ultimately, impairment of periosteal blood flow results in necrosis and bone resorption [8].

Immunocompromised conditions like diabetes mellitus impair the host immune system by suppressing it and limiting its response. The vascular changes in small vessels are compromised, resulting in low blood supply to tissues. This leads to a delayed healing mechanism, limiting immune response to prevailing pathogens that might cause infection. The above sequence of events significantly supports the incidence of osteomyelitis in the affected part [2].

Uncontrolled diabetes mellitus was the leading cause of maxillary osteomyelitis in our case. Based on a study, around 70% of maxillary osteomyelitis cases are associated with diabetes mellitus due to high blood glucose levels, which in turn impairs the immune system, altering the maxillary blood supply [9].

A higher degree of suspicion and prompt diagnosis is necessary when dealing with osteomyelitis of the jaw as it goes undiagnosed for a long time. Detailed clinical examinations, radiological and haematological investigations, and histopathological tests serve as different diagnostic modalities for the diagnosis of osteomyelitis. The sudden onset of infections is marked by high levels of neutrophils and leukocytes. A biopsy can be done to rule out any cancer differentials that might be mimicking osteomyelitis [10,11].

Other conditions that mimic osteomyelitis of the jaw are osteosarcoma, langerhans' cell histiocytosis, squamous cell carcinoma, and fibrous dysplasia. These conditions show bone destruction and periosteal reaction and make it challenging to diagnose osteomyelitis of the jaw [12].

The treatment plan should be aggressive and without any delay to avoid any further enhancement of the underlying disease. The treatment aims to strengthen the immune system, provide antibiotic cover, and surgical removal of dead and necrotic bone tissue, followed by surgical closure of operative site. The antibiotic cover may include amikacin, metronidazole, and linezolid [13]. The management spectrum involves simple non-invasive modalities and aggressive invasive surgical approaches. The non-invasive approach comprises hyperbaric oxygen therapy, antibiotics, and the use of bisphosphonates, as well as muscle relaxants. When non-surgical modalities seem ineffective in preserving bone structure, surgical methods like decortications, bone grafting, and partial or segmental resection are considered [14].

## Conclusions

Maxillary osteomyelitis is one of the rarest forms of osteomyelitis in today's time, thus making its diagnosis difficult. Limited literature exists on the epidemiology of maxillary osteomyelitis in India, therefore, highlighting the need for intensive research. Our case has emphasized the rarity and the incidence of cases of maxillary osteomyelitis due to diabetes mellitus. Osteomyelitis of the jaw in immunocompromised patients is a challenge to healthcare professionals in terms of diagnosis and management and, hence, requires a higher level of suspicion. Given its uncommon clinical features and overlap with other oral conditions, it can lead to severe infections affecting the cranial cavity and brain. Therefore, osteomyelitis of the jaw should be kept in mind while dealing with patients who complain of chronic pain after undergoing any dental extraction or trauma. Early diagnosis and prompt treatment with multifaceted modalities of management can significantly improve patient outcomes when dealing with patients with maxillary osteomyelitis with diabetes mellitus.
